# Bis{2-meth­oxy-6-[3-(methyl­amino)propyl­imino­meth­yl]phenolato}nickel(II) bis­(perchlorate)

**DOI:** 10.1107/S1600536808009562

**Published:** 2008-04-10

**Authors:** Yin-Ting He

**Affiliations:** a Zhoukou Vocational and Technical College, Zhoukou Henan 466600, People’s Republic of China

## Abstract

The asymmetric unit of the title compound, [Ni(C_12_H_18_N_2_O_2_)_2_](ClO_4_)_2_, consists of one-half of a centrosymmetric mononuclear Schiff base nickel(II) complex cation and one perchlorate anion. The Ni^II^ ion, lying on the inversion center, is coordinated by two N atoms and two O atoms from two Schiff base ligands, forming a square-planar geometry. The crystal packing is stabilized by N—H⋯O hydrogen bonds.

## Related literature

For related structures, see: Arıcı *et al.* (2005 ▶); Bian *et al.* (2004 ▶); Chen *et al.* (2008 ▶); Holm (1960 ▶); Ma, Gu *et al.* (2006 ▶); Ma, Lv *et al.* (2006 ▶); Ma, Wu *et al.* (2006 ▶); Ma *et al.* (2005 ▶); Skovsgaard *et al.* (2005 ▶); Zhao (2007 ▶); Zhu *et al.* (2004 ▶).
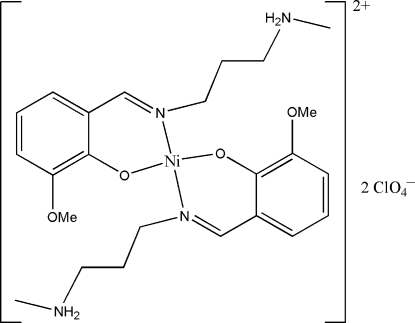


## Experimental

### 

#### Crystal data

[Ni(C_12_H_18_N_2_O_2_)_2_](ClO_4_)_2_
*M* *_r_* = 702.18
Orthorhombic, 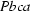

*a* = 13.557 (5) Å
*b* = 13.302 (5) Å
*c* = 17.371 (7) Å
*V* = 3133 (2) Å^3^
*Z* = 4
Mo *K*α radiation
μ = 0.85 mm^−1^
*T* = 298 (2) K
0.33 × 0.28 × 0.27 mm

#### Data collection

Bruker SMART CCD area-detector diffractometer
Absorption correction: multi-scan (*SADABS*; Sheldrick, 1996 ▶) *T*_min_ = 0.766, *T*_max_ = 0.802
16728 measured reflections
3276 independent reflections
2125 reflections with *I* > 2σ(*I*)
*R*_int_ = 0.042

#### Refinement

*R*[*F* ^2^ > 2σ(*F* ^2^)] = 0.055
*wR*(*F* ^2^) = 0.188
*S* = 1.04
3276 reflections
199 parameters
H-atom parameters constrained
Δρ_max_ = 0.97 e Å^−3^
Δρ_min_ = −0.55 e Å^−3^

### 

Data collection: *SMART* (Bruker, 1998 ▶); cell refinement: *SAINT* (Bruker, 1998 ▶); data reduction: *SAINT*; program(s) used to solve structure: *SHELXS97* (Sheldrick, 2008 ▶); program(s) used to refine structure: *SHELXL97* (Sheldrick, 2008 ▶); molecular graphics: *SHELXTL* (Sheldrick, 2008 ▶); software used to prepare material for publication: *SHELXL97*.

## Supplementary Material

Crystal structure: contains datablocks global, I. DOI: 10.1107/S1600536808009562/ci2578sup1.cif

Structure factors: contains datablocks I. DOI: 10.1107/S1600536808009562/ci2578Isup2.hkl

Additional supplementary materials:  crystallographic information; 3D view; checkCIF report

## Figures and Tables

**Table:** 

Ni1—O1	1.922 (3)
Ni1—N1	2.018 (3)

**Table:** 

O1^i^—Ni1—O1	180
O1—Ni1—N1	90.30 (14)
O1—Ni1—N1^i^	89.70 (13)
N1—Ni1—N1^i^	180

Symmetry code: (i) 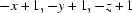.

**Table 2:** Hydrogen-bond geometry (Å, °)

*D*—H⋯*A*	*D*—H	H⋯*A*	*D*⋯*A*	*D*—H⋯*A*
N2—H2*A*⋯O1^i^	0.90	1.80	2.691 (4)	170
N2—H2*A*⋯O2^i^	0.90	2.44	2.929 (5)	114
N2—H2*B*⋯O4^ii^	0.90	2.23	3.075 (8)	157

Symmetry codes: (i) 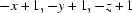; (ii) 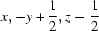.

**Fig. 1.**
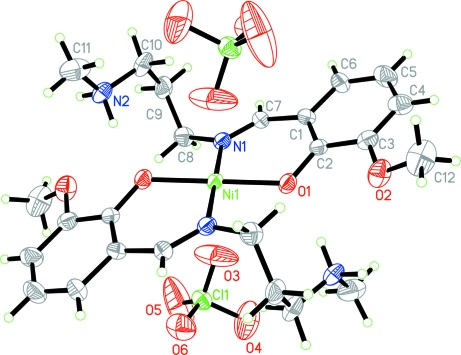
The molecular structure of the title compound, showing 30% probability displacement ellipsoids. Unlabelled atoms are related to labelled atoms by the symmetry operation (1 - x, 1 - y, 1 - z).

## supplementary crystallographic information

### Comment

Nickel(II) complexes with Schiff base ligands have been of great interest in coordination chemistry related to molecular structures and catalytical applications (Chen *et al.*, 2008; Holm, 1960; Arıcı *et al.*, 2005). Metal complexes derived from Schiff bases have been widely studied (Ma, Lv *et al.*, 2006; Ma, Gu *et al.*, 2006; Ma, Wu *et al.*, 2006; Ma *et al.*, 2005). However, the complexes derived from the Schiff base ligand 2-methoxy-6-[(3-methylaminopropylimino)methyl]phenol have never been reported. The author reports herein the title mononuclear nickel(II) complex.

The title compound consists of a centrosymmetric nickel(II) complex cation and two perchlorate anions (Fig. 1). The Ni^II^ ion, lying on the inversion center, is coordinated by two nitrogen atoms and two oxygen atoms from two Schiff base ligands, giving a square planar geometry. All the bond lengths and angles (Table 1) involving the Ni^II^ atom are within normal ranges, and comparable to values observed in other Schiff base nickel(II) complexes (Zhu *et al.*, 2004; Zhao, 2007; Bian *et al.*, 2004; Skovsgaard *et al.*, 2005). The N1—C8—C9—C10 and C9—C10—N2—C11 torsion angles are 55.0 (3) and 2.7 (3)°, respectively. The crystal packing is stabilized by N—H···O hydrogen bonds (Table 2).

### Experimental

*N*-Methylpropane-1,3-diamine (0.5 mmol, 44.0 mg) and 3-methoxysalicylaldehyde (0.5 mmol, 76.0 mg) were dissolved in methanol (30 ml). The mixture was stirred for 1 h to obtain a clear yellow solution. To the solution was added with stirring a methanol solution (20 ml) of nickel(II) perchlorate (0.5 mmol, 192.0 mg). After keeping the resulting solution in air for a few days, red block-shaped crystals were formed.

### Refinement

All H atoms were placed in geometrically idealized positions and constrained to ride on their parent atoms with C-H = 0.93–0.97 Å, N-H = 0.90 Å, and with *U*_iso_(H) = 1.2*U*_eq_(C,N) and 1.5*U*_eq_(methyl C).

### Crystal data

**Table:**

[Ni(C_12_H_18_N_2_O_2_)_2_](ClO_4_)_2_	*F*_000_ = 1464
*M**_r_* = 702.18	*D*_x_ = 1.489 Mg m^−^^3^
Orthorhombic, *P**b**c**a*	Mo *K*α radiation λ = 0.71073 Å
Hall symbol: -P 2ac 2ab	Cell parameters from 3048 reflections
*a* = 13.557 (5) Å	θ = 2.3–25.3º
*b* = 13.302 (5) Å	µ = 0.86 mm^−^^1^
*c* = 17.371 (7) Å	*T* = 298 (2) K
*V* = 3133 (2) Å^3^	Block, red
*Z* = 4	0.33 × 0.28 × 0.27 mm

### Data collection

**Table:**

Bruker SMART CCD area-detector diffractometer	3276 independent reflections
Radiation source: fine-focus sealed tube	2125 reflections with *I* > 2σ(*I*)
Monochromator: graphite	*R*_int_ = 0.042
*T* = 298(2) K	θ_max_ = 26.6º
ω scans	θ_min_ = 2.4º
Absorption correction: multi-scan(SADABS; Sheldrick, 1996)	*h* = −8→17
*T*_min_ = 0.766, *T*_max_ = 0.802	*k* = −16→15
16728 measured reflections	*l* = −19→21

### Refinement

**Table:**

Refinement on *F*^2^	Hydrogen site location: inferred from neighbouring sites
Least-squares matrix: full	H-atom parameters constrained
*R*[*F*^2^ > 2σ(*F*^2^)] = 0.055	*w* = 1/[σ^2^(*F*_o_^2^) + (0.0892*P*)^2^ + 4.466*P*] where *P* = (*F*_o_^2^ + 2*F*_c_^2^)/3
*wR*(*F*^2^) = 0.188	(Δ/σ)_max_ = 0.001
*S* = 1.04	Δρ_max_ = 0.97 e Å^−^^3^
3276 reflections	Δρ_min_ = −0.55 e Å^−^^3^
199 parameters	Extinction correction: SHELXL97 (Sheldrick, 2008), Fc^*^=kFc[1+0.001xFc^2^λ^3^/sin(2θ)]^-1/4^
Primary atom site location: structure-invariant direct methods	Extinction coefficient: 0.0023 (6)
Secondary atom site location: difference Fourier map	

### Special details

**Table:**

Geometry. All e.s.d.'s (except the e.s.d. in the dihedral angle between two l.s. planes) are estimated using the full covariance matrix. The cell e.s.d.'s are taken into account individually in the estimation of e.s.d.'s in distances, angles and torsion angles; correlations between e.s.d.'s in cell parameters are only used when they are defined by crystal symmetry. An approximate (isotropic) treatment of cell e.s.d.'s is used for estimating e.s.d.'s involving l.s. planes.
Refinement. Refinement of *F*^2^ against ALL reflections. The weighted *R*-factor *wR* and goodness of fit *S* are based on *F*^2^, conventional *R*-factors *R* are based on *F*, with *F* set to zero for negative *F*^2^. The threshold expression of *F*^2^ > σ(*F*^2^) is used only for calculating *R*-factors(gt) *etc*. and is not relevant to the choice of reflections for refinement. *R*-factors based on *F*^2^ are statistically about twice as large as those based on *F*, and *R*- factors based on ALL data will be even larger.

### Fractional atomic coordinates and isotropic or equivalent isotropic displacement parameters (Å^2^)

**Table:**

	*x*	*y*	*z*	*U*_iso_*/*U*_eq_	
Ni1	0.5000	0.5000	0.5000	0.0412 (3)	
Cl1	0.39978 (9)	0.29292 (9)	0.63654 (7)	0.0623 (4)	
O1	0.5954 (2)	0.5558 (2)	0.56985 (16)	0.0550 (8)	
O2	0.6861 (3)	0.7085 (3)	0.6382 (2)	0.0792 (11)	
O3	0.4646 (5)	0.3472 (5)	0.5938 (4)	0.178 (3)	
O4	0.4518 (5)	0.2905 (5)	0.7084 (4)	0.169 (3)	
O5	0.3852 (5)	0.1987 (5)	0.6207 (7)	0.260 (6)	
O6	0.3118 (3)	0.3474 (4)	0.6470 (3)	0.1112 (15)	
N1	0.6046 (3)	0.4103 (3)	0.45407 (18)	0.0497 (8)	
N2	0.4565 (3)	0.4199 (3)	0.2818 (2)	0.0604 (10)	
H2A	0.4351	0.4211	0.3308	0.072*	
H2B	0.4363	0.3615	0.2608	0.072*	
C1	0.7430 (4)	0.5058 (3)	0.5061 (2)	0.0549 (11)	
C2	0.6908 (3)	0.5687 (3)	0.5551 (2)	0.0495 (10)	
C3	0.7427 (4)	0.6482 (4)	0.5911 (2)	0.0586 (11)	
C4	0.8418 (4)	0.6607 (4)	0.5796 (3)	0.0715 (14)	
H4	0.8747	0.7132	0.6040	0.086*	
C5	0.8935 (4)	0.5960 (5)	0.5320 (4)	0.0797 (16)	
H5	0.9611	0.6044	0.5251	0.096*	
C6	0.8456 (4)	0.5204 (4)	0.4957 (3)	0.0715 (15)	
H6	0.8806	0.4774	0.4634	0.086*	
C7	0.6969 (3)	0.4249 (3)	0.4641 (2)	0.0563 (11)	
H7	0.7391	0.3780	0.4418	0.068*	
C8	0.5803 (4)	0.3206 (3)	0.4076 (3)	0.0633 (12)	
H8A	0.5102	0.3074	0.4118	0.076*	
H8B	0.6150	0.2630	0.4286	0.076*	
C9	0.6073 (4)	0.3317 (4)	0.3228 (3)	0.0731 (15)	
H9A	0.6786	0.3348	0.3187	0.088*	
H9B	0.5858	0.2716	0.2959	0.088*	
C10	0.5648 (4)	0.4214 (4)	0.2820 (3)	0.0713 (14)	
H10A	0.5874	0.4823	0.3072	0.086*	
H10B	0.5886	0.4226	0.2294	0.086*	
C11	0.4092 (6)	0.5042 (4)	0.2391 (4)	0.099 (2)	
H11A	0.4323	0.5671	0.2594	0.149*	
H11B	0.3389	0.5002	0.2450	0.149*	
H11C	0.4260	0.4997	0.1856	0.149*	
C12	0.7201 (6)	0.8047 (4)	0.6537 (4)	0.113 (2)	
H12A	0.7755	0.8010	0.6880	0.169*	
H12B	0.6684	0.8433	0.6771	0.169*	
H12C	0.7400	0.8363	0.6065	0.169*	

### Atomic displacement parameters (Å^2^)

**Table:**

	*U*^11^	*U*^22^	*U*^33^	*U*^12^	*U*^13^	*U*^23^
Ni1	0.0409 (4)	0.0501 (4)	0.0326 (4)	−0.0002 (3)	0.0013 (3)	−0.0056 (3)
Cl1	0.0584 (7)	0.0553 (6)	0.0733 (8)	−0.0012 (5)	0.0005 (6)	0.0103 (5)
O1	0.0486 (17)	0.073 (2)	0.0437 (15)	−0.0064 (14)	0.0031 (13)	−0.0133 (14)
O2	0.082 (3)	0.078 (2)	0.078 (2)	−0.0215 (19)	0.0081 (19)	−0.0288 (19)
O3	0.144 (5)	0.192 (6)	0.197 (6)	0.044 (4)	0.085 (5)	0.123 (5)
O4	0.184 (6)	0.177 (6)	0.146 (5)	0.022 (5)	−0.090 (5)	0.013 (4)
O5	0.145 (6)	0.115 (5)	0.519 (17)	0.000 (4)	−0.079 (7)	−0.150 (8)
O6	0.081 (3)	0.119 (4)	0.133 (4)	0.035 (3)	0.008 (3)	0.024 (3)
N1	0.057 (2)	0.0515 (19)	0.0406 (18)	0.0053 (16)	−0.0010 (15)	−0.0024 (14)
N2	0.078 (3)	0.061 (2)	0.0424 (19)	−0.008 (2)	0.0029 (18)	−0.0111 (17)
C1	0.047 (2)	0.069 (3)	0.049 (2)	0.009 (2)	−0.0043 (19)	0.009 (2)
C2	0.046 (2)	0.063 (3)	0.039 (2)	0.0010 (19)	−0.0043 (17)	0.0042 (18)
C3	0.062 (3)	0.067 (3)	0.046 (2)	−0.009 (2)	−0.004 (2)	0.000 (2)
C4	0.059 (3)	0.083 (4)	0.072 (3)	−0.016 (3)	−0.012 (3)	0.008 (3)
C5	0.044 (3)	0.100 (4)	0.095 (4)	−0.006 (3)	−0.007 (3)	0.010 (4)
C6	0.047 (3)	0.094 (4)	0.073 (3)	0.012 (3)	0.002 (2)	0.005 (3)
C7	0.054 (3)	0.066 (3)	0.049 (2)	0.015 (2)	0.000 (2)	0.001 (2)
C8	0.073 (3)	0.049 (2)	0.069 (3)	0.008 (2)	−0.004 (2)	−0.006 (2)
C9	0.071 (3)	0.083 (4)	0.065 (3)	0.005 (3)	0.004 (2)	−0.034 (3)
C10	0.082 (4)	0.082 (4)	0.049 (3)	−0.019 (3)	0.016 (2)	−0.019 (2)
C11	0.140 (6)	0.083 (4)	0.075 (4)	0.011 (4)	−0.027 (4)	0.002 (3)
C12	0.159 (7)	0.066 (4)	0.114 (5)	−0.024 (4)	0.037 (5)	0.007 (4)

### Geometric parameters (Å, °)

**Table:**

Ni1—O1^i^	1.922 (3)	C3—C4	1.369 (7)
Ni1—O1	1.922 (3)	C4—C5	1.385 (8)
Ni1—N1	2.018 (3)	C4—H4	0.93
Ni1—N1^i^	2.018 (3)	C5—C6	1.353 (8)
Cl1—O5	1.298 (6)	C5—H5	0.93
Cl1—O3	1.358 (5)	C6—H6	0.93
Cl1—O6	1.408 (4)	C7—H7	0.93
Cl1—O4	1.434 (5)	C8—C9	1.525 (7)
O1—C2	1.329 (5)	C8—H8A	0.97
O2—C3	1.378 (6)	C8—H8B	0.97
O2—C12	1.386 (6)	C9—C10	1.502 (8)
N1—C7	1.278 (5)	C9—H9A	0.97
N1—C8	1.477 (5)	C9—H9B	0.97
N2—C10	1.469 (7)	C10—H10A	0.97
N2—C11	1.489 (6)	C10—H10B	0.97
N2—H2A	0.90	C11—H11A	0.96
N2—H2B	0.90	C11—H11B	0.96
C1—C2	1.389 (6)	C11—H11C	0.96
C1—C6	1.416 (7)	C12—H12A	0.96
C1—C7	1.443 (6)	C12—H12B	0.96
C2—C3	1.416 (6)	C12—H12C	0.96
			
O1^i^—Ni1—O1	180	C4—C5—H5	120.1
O1^i^—Ni1—N1	89.70 (13)	C5—C6—C1	120.9 (5)
O1—Ni1—N1	90.30 (14)	C5—C6—H6	119.5
O1^i^—Ni1—N1^i^	90.30 (14)	C1—C6—H6	119.5
O1—Ni1—N1^i^	89.70 (13)	N1—C7—C1	127.3 (4)
N1—Ni1—N1^i^	180	N1—C7—H7	116.3
O5—Cl1—O3	119.7 (6)	C1—C7—H7	116.3
O5—Cl1—O6	113.3 (4)	N1—C8—C9	113.3 (4)
O3—Cl1—O6	110.2 (3)	N1—C8—H8A	108.9
O5—Cl1—O4	103.7 (6)	C9—C8—H8A	108.9
O3—Cl1—O4	99.8 (5)	N1—C8—H8B	108.9
O6—Cl1—O4	108.4 (4)	C9—C8—H8B	108.9
C2—O1—Ni1	125.7 (2)	H8A—C8—H8B	107.7
C3—O2—C12	117.8 (4)	C10—C9—C8	116.1 (4)
C7—N1—C8	114.5 (4)	C10—C9—H9A	108.3
C7—N1—Ni1	123.0 (3)	C8—C9—H9A	108.3
C8—N1—Ni1	122.5 (3)	C10—C9—H9B	108.3
C10—N2—C11	114.9 (5)	C8—C9—H9B	108.3
C10—N2—H2A	108.5	H9A—C9—H9B	107.4
C11—N2—H2A	108.5	N2—C10—C9	111.9 (4)
C10—N2—H2B	108.5	N2—C10—H10A	109.2
C11—N2—H2B	108.5	C9—C10—H10A	109.2
H2A—N2—H2B	107.5	N2—C10—H10B	109.2
C2—C1—C6	119.8 (4)	C9—C10—H10B	109.2
C2—C1—C7	122.6 (4)	H10A—C10—H10B	107.9
C6—C1—C7	117.6 (4)	N2—C11—H11A	109.5
O1—C2—C1	122.4 (4)	N2—C11—H11B	109.5
O1—C2—C3	119.7 (4)	H11A—C11—H11B	109.5
C1—C2—C3	117.9 (4)	N2—C11—H11C	109.5
C4—C3—O2	124.2 (4)	H11A—C11—H11C	109.5
C4—C3—C2	120.9 (5)	H11B—C11—H11C	109.5
O2—C3—C2	114.8 (4)	O2—C12—H12A	109.5
C3—C4—C5	120.6 (5)	O2—C12—H12B	109.5
C3—C4—H4	119.7	H12A—C12—H12B	109.5
C5—C4—H4	119.7	O2—C12—H12C	109.5
C6—C5—C4	119.8 (5)	H12A—C12—H12C	109.5
C6—C5—H5	120.1	H12B—C12—H12C	109.5

Symmetry codes: (i) −*x*+1, −*y*+1, −*z*+1.

### Hydrogen-bond geometry (Å, °)

**Table:**

*D*—H···*A*	*D*—H	H···*A*	*D*···*A*	*D*—H···*A*
N2—H2A···O1^i^	0.90	1.80	2.691 (4)	170
N2—H2A···O2^i^	0.90	2.44	2.929 (5)	114
N2—H2B···O4^ii^	0.90	2.23	3.075 (8)	157

Symmetry codes: (i) −*x*+1, −*y*+1, −*z*+1; (ii) *x*, −*y*+1/2, *z*−1/2.
